# Hematopoietic monoamine oxidase A deficiency exacerbates neuroinflammation and demyelination in female but not male mice with experimental autoimmune encephalomyelitis

**DOI:** 10.3389/fimmu.2026.1829375

**Published:** 2026-06-30

**Authors:** Alessandro G. Salerno, Amarylis C. B. A. Wanschel, Giovana Y. A. Mascoli, Seonwook Kim, Elena Boudyguina, Reto Asmis

**Affiliations:** 1 Department of Internal Medicine, Wake Forest School of Medicine, Winston-Salem, NC, United States; 2 Department of Basic Pharmaceutical Sciences, Fred Wilson School of Pharmacy, High Point University, High Point, NC, United States; 3 Department of Neuroscience, Wanek School of Natural Sciences, High Point University, High Point, NC, United States

**Keywords:** microglia, monoamine oxidase, monocyte-derived macrophages (MDM), redox signaling, sex differences

## Abstract

**Introduction:**

Monoamine oxidase A (Mao A) is a mitochondrial enzyme responsible for the degradation of monoaminergic neurotransmitters. While pharmacological inhibition of Mao A has been reported to improve outcomes in experimental autoimmune encephalomyelitis (EAE), the role of Mao A in immune cells on disease progression was unknown.

**Methods:**

To address this question, we generated mice with conditional Mao A deletion restricted to hematopoietic cells (Mao A_Leuko_^-/-^), including circulating immune cells, and induced EAE by immunizing the mice with the MOG35-55 peptide. Disease severity, ataxia, inflammation, and demyelination were assessed.

**Results and discussion:**

Mao A_Leuko_^-/-^ female mice exhibited significantly accelerated weight loss, worsened ataxia, and a 2.2-fold increase in EAE severity compared with controls. Histological analyses revealed increased inflammation and enhanced demyelination in the lumbar spinal cords of Mao A_Leuko_^-/-^ mice. Immunohistochemistry revealed increased IBA-1^+^ and CD68^+^ staining in the spinal cord, consistent with enhanced microglia and macrophage accumulation and activation and exacerbated neuroinflammation. However, no differences in CD3^+^ T-cells levels were observed between female Mao A_Leuko_^-/-^ and Vav-i Cretg/wt EAE mice. These effects were restricted to female mice. No significant differences were observed in disease course or pathology between male Mao A_Leuko_^-/-^ and control mice. This study identified hematopoietic Mao A as a novel critical regulator of neuroimmune crosstalk, with loss of Mao A function exacerbating inflammation, demyelination, and neurological deficits in female but not male EAE mice.

## 1 Introduction

Multiple sclerosis (MS) is a complex disease of unclear, multifactorial etiology (1) affecting the central nervous system (CNS). MS lesions are defined by inflammation, gliosis, demyelination, and/or neurodegeneration (2, 3) and can affect both the white and gray matter (WM and GM) of the CNS (4, 5). Their distribution and composition vary between and within patients (5), and their localization often reflects the clinical symptoms, contributing to high clinical heterogeneity (6). MS affects more women than men, with a gender ratio approaching 3:1 (7). Women and men exhibit well-established differences in both innate and adaptive immunity, which contribute to differential susceptibility to autoimmune diseases (8). In the central nervous system, microglia display sex-specific transcriptional and functional profiles, influencing neuroinflammatory processes and disease outcomes (9, 10). Consistent with this, multiple sclerosis and its animal model, experimental autoimmune encephalomyelitis (EAE), show a strong female bias and are modulated by sex-dependent neuroimmune mechanisms (11).

MS is thought to be an autoimmune-mediated CNS disorder where both adaptive and innate immune systems are involved to different extents during the disease (12–14). The early, relapsing-remitting (RR) stage of MS is associated with antigen-specific T and B cell-mediated adaptive immune responses, whereas the progressive phase of the disease is associated with innate immune responses characterized by chronic inflammation and microglial activation (15). Experimental autoimmune encephalomyelitis (EAE), a well-established animal model that replicates MS in humans (16, 17), is characterized by myelin-specific T cells that are peripherally activated in secondary organs, such as lymph nodes, and migrate towards the CNS interfaces to get reactivated by antigen-presenting cells (APC) and clonally expand to effectively carry out their actions (18, 19). Besides T cells, resident microglia, CNS-associated macrophages (CAMs, found in interfaces such as the perivascular space or the meninges), and infiltrating monocyte-derived macrophages (MDM), along with neutrophils, are associated with the development of the MS/EAE pathology (20). All these myeloid populations are known to participate in both beneficial and detrimental processes during the development of MS pathology (21). This dichotomic behavior can be attributed to the distinct activation states of microglia and macrophages, e.g., proinflammatory versus inflammation-resolving phenotypes (classically activated, alternatively activated, or disease-associated activated) (22). Thus, modulation of microglia/macrophage response critically influences the outcome of EAE and shapes the balance between ongoing tissue injury and remission (23, 24).

Monoamines are crucial for bidirectional interactions between the immune and nervous systems due to their ability to bind to cell receptors in both systems (25). They encompass three main types of neurotransmitters: dopamine (DA), noradrenaline (NA), and serotonin (5-hydroxytryptamine, 5HT) and are produced by monoaminergic neurons, located within the brainstem (26). However, neurotransmitter levels are significantly reduced in the CNS of mice with EAE (27), suggesting they may play a critical role in the pathogenesis of MS and EAE. These alterations in concentration are not only quantitative findings but also carry functional implications, since monoamines actively modulate immune and glial responses. For example, NA exerts potent anti-inflammatory and neuroprotective effects, acting mainly on microglia and astrocytes (28). 5HT regulates both the innate and adaptive immune system, including by driving macrophage polarization towards inflammation-resolving M2-like phenotypes (29, 30). NA, dopamine and 5HT are degraded by monoamine oxidase A (Mao A), an FAD-containing enzyme tightly bound to the outer mitochondrial membrane that catalyzes the oxidative deamination of primary, secondary and tertiary amines, including dietary biogenic amines entering and accumulating in the blood stream, such as tyramine, and endogenous neurotransmitters, to the corresponding imines, which are then hydrolyzed to the corresponding aldehydes or ketones (31). Mao A is expressed predominantly in specific regions of the CNS and peripheral tissues (31). In the brain, Mao A has a broad tissue distribution and is highly expressed in noradrenergic neurons (32, 33).

Mao A is also expressed in immune cells, particularly in monocytes and macrophages (34, 35) as well as microglia (36). Importantly, recruitment of peripheral leukocytes through the blood-brain and blood-cerebrospinal fluid barriers into the CNS is a hallmark of MS (37) and these immune cells may contribute to the depletion of monoaminergic neurotransmitters in the CNS associated with MS. Two studies showed that the Mao inhibitor phenelzine improves the functional outcomes in mice with EAE, a mouse model of human MS. The drug was effective when provided prior to the onset of clinical symptoms as well as in mice already exhibiting EAE symptoms (38, 39). In this study we therefore examined whether genetic deletion of Mao A activity in hematopoietic cells of EAE mice, including circulating immune cells, would equally reduce EAE disease severity and physical impairments of these mice. Given that monoaminergic neurotransmitters such as serotonin and dopamine play important roles in modulating inflammatory responses and cytokine production (40), and considering the well-established sex differences in both innate and adaptive immunity (8, 10, 11, 41), we predicted that alterations in Mao A activity in hematopoietic cells may result in sex-dependent effects on neuroimmune interactions and EAE pathogenesis.

## 2 Materials and methods

### 2.1 Reagents

All reagents used in this study and their sources are listed in **Table 1**.

**Table 1:** Reagents antibodies are listed with their final dilutions.

Reagent	Clone / catalog #	Dilution	Source
DAPI	D9542	1:2,000	Sigma-Aldrich, St. Louis, MO, USA
Eosin-Y	7111		Richard-Allan Scientific, Kalamazoo, MI, USA
FBS	10082-147		Gibco, Waltham, MA, USA
anti-CD3	ab11089	1:1,000	Abcam, Cambridge, MA, USA
anti-CD68	MCA1957	1:1,000	Bio-Rad, Hercules, CA, USA
anti-IBA-1	019-19741	1:1,000	FUJIFILM Wako Pure Chemical Corporation, USA
Hematoxylin	7211		Richard-Allan Scientific, Kalamazoo, MI, USA
HydroGel®	70-01-5022		ClearH2O, Portland, ME, USA
MOG_35–55_ peptide	EK-2110		Hooke Laboratories, Lawrence, MA, USA
Laemmli Sample Buffer	161-0747		Bio-Rad, Hercules, CA, USA
Lithium carbonate	50-318-47		Electron Microscopy Sciences, Hatfield, PA, USA
Luxol Fast Blue	12218A		Newcomer Supply, Waunakee, WI, USA
β-Mercaptoethanol	161-0710		Bio-Rad, Hercules, CA, USA
Paraformaldehyde (PFA)	28906		Thermo Scientific, Rockford, IL, USA
Pertussis toxin	EK-2110		Hooke Laboratories, Lawrence, MA, USA
PBS (pH 7.4)	10010023		Thermo Scientific Rockford, IL, USA
Restore™ PLUS Western Blot Stripping Buffer	46430		Thermo Scientific, Rockford, IL, USA
RIPA buffer	89901		Thermo Scientific, Rockford, IL, USA
Rabbit anti-Mao A	ab126751	1:1,000	Abcam, Waltham, MA, USA
Rabbit anti-Mao B	ab137778	1:1,000	Abcam, Waltham, MA, USA
Rabbit anti-β-actin-HRP	#13E5	1:2,000	Cell Signaling Technology, Danvers, MA, USA,
anti-Rabbit IgG	#7074S	1:2,000	Cell Signaling Technology, Danvers, MA, USA
Goat anti-Rabbit Alexa-Fluor 488	A11034	1:1,000	Invitrogen, Carlsbad, CA, USA
Donkey Anti-rat, Alexa Fluor 594	A21209	1:1,000	Invitrogen, Carlsbad, CA, USA

### 2.2 Animals and diet

Male and Female C57BL/6J (stock number 000664) and Vav-iCre (stock number 018968) mice were obtained from Jackson Laboratory (Bar Harbor, ME, USA). Conditional Mao A knockout mice (Mao A^flox/flox^) were generated by Applied StemCell (Milpitas, CA, USA) using CRISPR/Cas9 in a C57BL/6J genetic background [36]. Mao A^flox/flox^ mice were crossed with Vav-iCre^tg/wt^ mice, which express the improved Cre recombinase (iCre) under the control of the mouse Vav 1 oncogene (Vav) promoter, to generate Vav-iCre^tg/wt^Mao A^flox/flox^ (Mao A_Leuko_^-/-^) mice. Mice from both strains were co-housed in the same cages, which reduced cage- and handling-associated bias. Male and female Mao A_Leuko_^-/-^ and Vav-iCre^tg/wt^ control mice were maintained in colony cages on a 12-h light/12-h dark cycle and fed a formulated mouse chow diet (Prolab^®^ RMH 3000, LabDiet, Richmond, Indiana, USA). At 8-weeks of age, all mice were randomized into different experimental groups and switched to a defined low-fat maintenance diet (MD, 3.93 kcal/g, 20% protein, 7.2% fat, 61.6% carbohydrates, F4031, Bioserv, Flemington, NJ, USA) and maintained on MD until the end of the experiment. All studies were performed in accordance with the guidelines and regulations of and with the approval of the Wake Forest School of Medicine Institutional Animal Care and Use Committee.

### 2.3 White blood cell isolation and western blot analysis of Mao A expression

Blood samples were collected from the submandibular vein of Mao A_Leuko_^-/-^ and Vav-iCre^tg/wt^ control mice randomly (n = 4) and washed with RoboSep buffer (Stem Cell Technologies, Vancouver, BC, Canada). After centrifugation at 300 × *g* for 5 min at RT, the supernatant was removed and cells were treated with ice-cold red blood cell (RBC) lysis buffer for 10 min, washed, and centrifuged to create a white blood cell (WBC) pellet.

WBC pellets were lysed in RIPA buffer (Thermo Scientific, Rockford, IL, USA) supplemented with protease inhibitor and phosphatase inhibitor cocktail (Thermo Scientific, Rockford, IL, USA). Protein lysates were boiled with Laemmli Sample Buffer (Bio-Rad, Hercules, CA, USA) containing 355 mM β-mercaptoethanol, and aliquots with equal amounts of protein were loaded on 7.5% SDS-PAGE gels (Bio-Rad, Hercules, CA, USA) and run. Subsequently, separated proteins were transferred onto PVDF membranes (Bio-Rad, Hercules, CA, USA). PVDF membrane blots were blocked in 5% BSA in TBS-T (20 mM Tris, 0.16 M NaCl, and 0.10% Tween-20, pH 7.4) for 1 h at RT, and incubated overnight at 4 °C with rabbit anti-Mao A antibodies (1:1, 000, ab126751, Abcam, Waltham, MA, USA) in 5% BSA in TBS-T. Anti-rabbit IgG (#7074S, Cell Signaling Technology, Danvers, MA, USA) was used as secondary antibodies. Blots were stripped by incubating in Restore™ PLUS Western Blot Stripping Buffer (Thermo Scientific, Rockford, IL, USA) and reprobed with a rabbit anti-β-actin-HRP antibody (1:1, 000, #13E5, Cell Signaling Technology, Danvers, MA, USA). The bands were detected by chemiluminescence (Radiance Plus Femtogram HRP substrate, Azure Biosystems, Dublin, CA, USA) on an Azure 600 Imager (Azure Biosystems, Dublin, CA, USA).

### 2.4 EAE induction

EAE was induced using a commercially available EAE induction kit (EK-2110, Hooke Laboratories, Lawrence, MA, USA) according to the manufacturer’s instructions. Briefly, mice were injected subcutaneously into the upper and lower backs with MOG_35–55_ peptides emulsified with complete Freund’s adjuvant (CFA). Additionally, mice received 200 ng of pertussis toxin (PTX, kit component) intraperitoneally (i.p.) at 2 h and 24 h after the MOG_35–55_/CFA injection. Mice were weighed and clinically scored daily for 30 days. At the end of the experiment, mice were sedated with isoflurane (4% for induction, 2% for maintenance, delivered in oxygen via a precision vaporizer) and subsequently euthanized by transcardial perfusion with phosphate-buffered saline (PBS, pH 7.4), followed by 4% paraformaldehyde (PFA). The CNS was surgically removed as described in (42)and fixed with 4% PFA in PBS.

### 2.5 EAE disease and ataxia scoring

EAE disease severity and Ataxia were assessed as described by Forsthuber and colleagues (43). EAE disease severity was scored on a scale of *0-5* (*0*: no clinical disease; *1*: flaccid tail; *2*: partial hind-limb paralysis; *3*: total hind-limb paralysis; *4*: front and hind-limb paralysis; *5*: moribund or dead). Ataxia was assessed and scored on a scale of *0–3* using the following four tests: Ledge test (*0*: balanced and graceful; *1*: loses footing but still shows coordination; *2*: cannot use hind legs well, slipping but balanced; *3*: legs shaking significantly, not graceful). Hindlimb clasping (*0*: hind legs move outward; *1*: 50% of the 10 s one hind limb is moved inward; *2*: 50% of the 10 s both hind limbs are moved inward; *3*: >50% of the 10 s, both hind limbs are near the abdomen). Gait (*0*: normal movement; *1*: slight tremor or waddle during movement; *2*: hind legs are shaking during movement, severe tremor; *3*: hind legs are dragging and abdomen near the floor). Kyphosis (*0*: flat back; *1*: initial curvature of the back but able to flatten while moving; *2*: consistent mild curvature of the back; *3*: consistent severe curvature of the back). The ataxia score was calculated as the sum of scores from all four tests. Mice that were scored at 3 or higher were provided with supplemental hydration-hydrogels HydroGel^®^ (ClearH2O, Portland, ME, USA) to prevent dehydration. Two mice died in the Mao A_Leuko_^-/-^ group during the peak of the disease between days 14 and 20. To prevent data bias, dead mice were assigned the last weight loss before they died and the maximum score for EAE and ataxia severity for the remaining days of the study (44).

### 2.6 Histological analysis

Spinal cords were harvested from each group at 30 days post-immunization for pathological assessment. Following transcardial perfusion with 4% PFA in PBS and immersion in the same fixative, tissues were carefully dissected. Formalin-fixed paraffin-embedded mouse lumbar spinal cords were sectioned at 5 µm thickness, adhered to charged slides (Thermo Scientific, Waltham, MA, USA) and allowed to dry overnight before staining. Slides were heated, deparaffinized in xylene, hydrated in 100% and 95% alcohol, and then stained with Hematoxylin (Richard-Allan Scientific, #7211, Kalamazoo, MI, USA) and Eosin-Y (Richard-Allan Scientific, #7111, Kalamazoo, MI, USA) for assessment of inflammation. For the assessment of (de)myelination, a second set of adjacent sections was stained for 2 h at 60 °C with 0.1% alcoholic Luxol Fast Blue (LFB) stain (Newcomer Supply, #12218A, Waunakee, WI, USA) under a tight seal. After LFB staining, slides were quickly dipped into 95% alcohol to remove excess stain, rinsed in distilled water, dipped five times into 0.05% lithium carbonate, and then into 70% alcohol (5–7 s) until decoloring of the cortical grey matter (GM), whilst white matter (WM) remained blue. The latter step was performed carefully and was checked microscopically to ensure homogeneity of the LFB staining. The differentiation steps were repeated if needed. The sections were then rinsed in distilled water before mounting the coverslip. All steps were completed in an automatic stainer (Gemini AS, Thermo Scientific, Waltham, MA, USA).

### 2.7 Inflammation and demyelination scores

Quantification of neuropathological features, although present both in the brain and spinal cord sections, was performed in the spinal cords because it is highly reproducible and well-established in the literature (45). Semi-quantitative histological evaluations and scoring were performed in a blinded fashion after lumbar spinal cords were scanned with a NanoZoomer S360 digital slide scanner (Hamamatsu Corporation, Bridgewater, NJ, USA) and images were processed using OlyVIA software (Olympus Corporation, Tokyo, Japan). Inflammation was scored in a blinded manner on H&E sections. For each section, the entire spinal cord cross-section was outlined as a Region of-Interest (ROI). H&E images were analyzed in ImageJ (v2., https://imagej.net/software/fiji/, accessed on 28 August 2025) (46). Color separation was performed with Color Deconvolution (“H&E 2” vectors) to obtain the hematoxylin channel. Within each cord ROI, cells-rich regions were segmented by automatic thresholding (e.g., Otsu; dark objects) (47). Area Fraction (%) was recorded as the hematoxylin-positive area divided by the total cord ROI area. Three lumbar sections per mouse were quantified and averaged to produce a single value per animal for statistical analysis. For the quantitation of changes in myelination, images of LFB-stained tissue sections were processed and analyzed using publicly available ImageJ software (version 2.0.0/1.52p, https://imagej.net/ij/, accessed on 10 June 2025). First, all micrographs were processed into grey-scale 8-bit images. Second, the image was prepared for thresholding into a binary black and white pixel image. To this end, within the ‘Binary Options’ menu the ‘Black background’ option was checked to ensure that black pixels represented the myelin when a value reaches the set threshold. Threshold values were set at a level that best matched the blue intensity of the LFB image, to retain as many true pixels representing myelin as possible. The contrast in the black and white image was visually equated with the corresponding LFB image. This resulted in a black and white binary image with either white pixels (value “240”) or black pixels (value “0”) (48, 49). In the black and white binary images, ROIs were manually drawn on the spinal cord border, on three different sections excluding (large) blood vessels, tissue folds or other irregularities. Results are reported as % myelin-positive areas per total section area, as described in (49).

### 2.8 Lumbar spinal cord immunofluorescence staining and imaging

Paraffin-embedded lumbar spinal cords were deparaffinized and cut cross-sectionally at a thickness of 5 µm. Antigen unmasking was performed by microwaving the slides twice for 10 min in citrate buffer solution (pH=6) (Sigma-Aldrich, St. Louis, MO, USA). Sections were then blocked for 1 h at RT with 10% normal goat serum Sigma-Aldrich, St. Louis, MO, USA), followed by overnight incubation at 4 °C with the primary antibodies directed against ionized calcium-binding adaptor molecule 1 (IBA1 019-19741, FUJIFILM Wako Pure Chemical Corporation, Richmond, VA, USA). Sections then incubated in Goat anti-rabbit Alexa-Fluor 488-labeled antibody (A11034, Invitrogen, Carlsbad, CA, USA) and washed with PBS containing DAPI (D9542, 1 µg/mL, Sigma-Aldrich, St. Louis, MO, USA). For the identification of macrophages and microglia, three IBA1-stained cross sections of the lumbar segments from the spinal cord of each mouse were imaged at 10x magnification. ImageJ software (version 2.0.0/1.52p, https://imagej.net/ij/, accessed on 20 August 2025) was used to analyze the signal intensity and calculate the areas occupied by the positively stained cells.

To define macrophages and microglia, sections were blocked for 1 h at RT with 10% normal goat serum Sigma-Aldrich, St. Louis, MO, USA), followed by overnight incubation at 4 °C with the primary antibody directed against CD68 (anti-CD68, MCA1957, Bio-Rad, Hercules, CA, USA). Sections were subsequently incubated with Alexa Fluor™ Plus 488–conjugated secondary antibodies Goat anti-rabbit, A11034, Invitrogen, Carlsbad, CA, USA) and washed with PBS containing DAPI (D9542, 1 μg/mL, Sigma-Aldrich, St. Louis, MO, USA). For the quantitation of macrophage and microglia recruitment and accumulation, CD68-stained cross-sections of the lumbar spinal cord from each mouse were imaged at 10× magnification. ImageJ software (version 2.0.0/1.52p, https://imagej.net/ij/accessed on 25 May 2026) was used to quantify the percentage of CD68^+^ area per section. A consistent intensity threshold was applied across all images to exclude background staining and ensure comparability between samples.

To identify T-cells, adjacent sections were blocked for 1 h at RT with 10% normal donkey serum Sigma-Aldrich, St. Louis, MO, USA) and incubated with a rat anti-mouse CD3 primary antibody (ab11089, Abcam, Cambridge, MA, USA), followed by incubation with donkey anti-rat IgG (H+L), Alexa Fluor 594-conjugated secondary antibody (A-21209, Invitrogen, Carlsbad, CA, USA). Sections were counterstained with DAPI and imaged as described above. Quantification of CD3^+^ cells was performed using ImageJ software (version 2.0.0/1.52p, https://imagej.net/ij/, accessed on 23 May 2026) by measuring the number of CD3^+^ cells per area within the lumbar spinal cord.

### 2.9 Statistical analyses

Mean values between the two experimental groups were compared by an unpaired two-tailed Student t-test (SigmaPlot 15). Unless stated otherwise, data are expressed as mean ± standard error of the mean (SEM). p < 0.05 was set as the statistical significance level.

## 3 Results

### 3.1 Hematopoietic Mao A deficiency exacerbates EAE-associated weight loss

**Figure 1:**
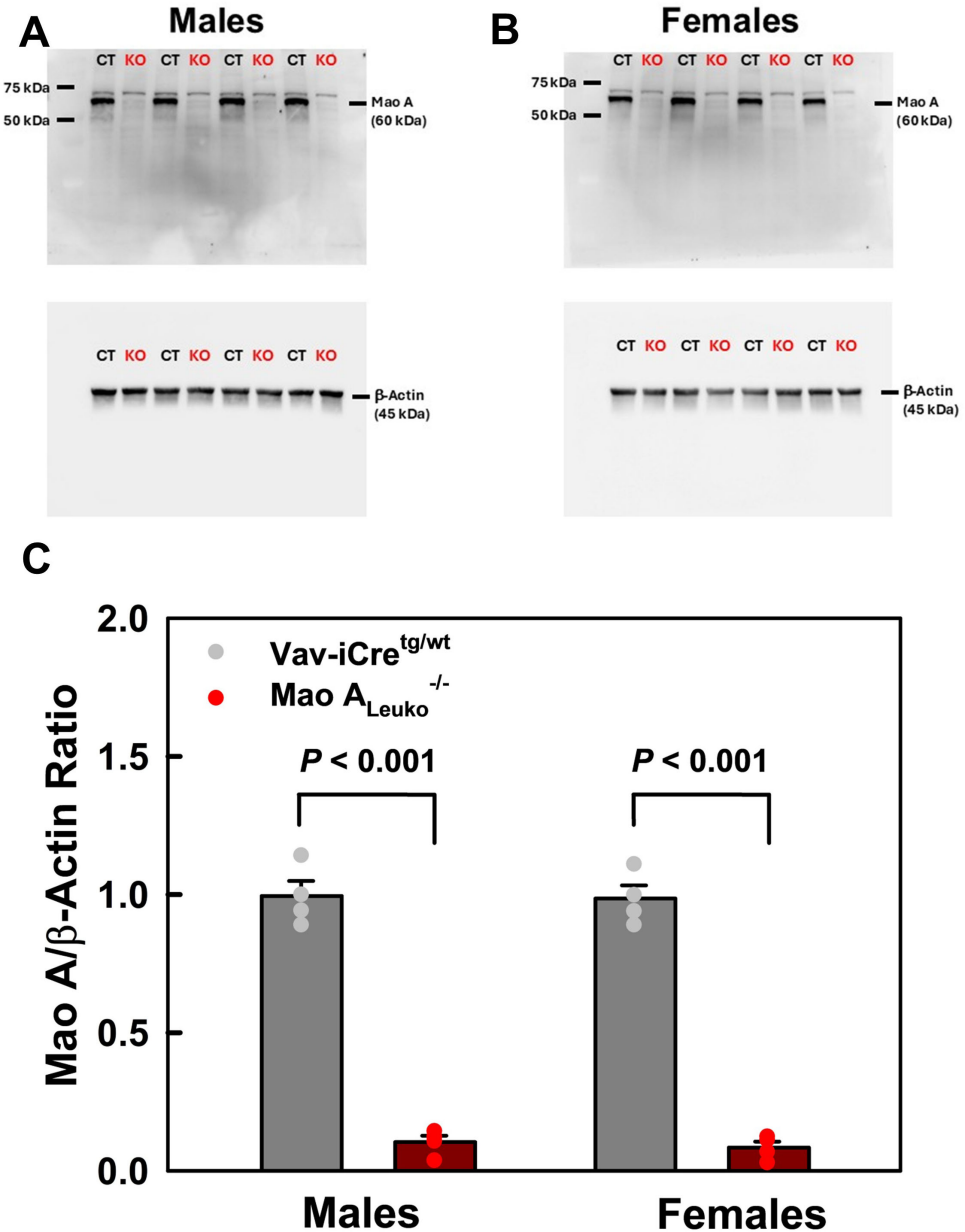
Mao A Expression Levels in White Blood Cells (WBC) Isolated From Vav-iCre^tg/wt^ and Mao A_Leuko_^-/-^ Mice. Blood samples were collected from the submandibular veins of four randomly selected male and female Vav-iCre^tg/wt^ (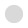, n = 4) and Mao A_Leuko_^-/-^ (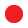, n =4) mice, washed and treated with ice-cold red blood cell (RBC) lysis buffer to create a WBC pellet. Mao A expression in each of these WBC pellets was assessed by Western blot analysis **(A, B)**. The blot was then stripped and reprobed with β-actin as described in detail under “Materials and Methods”. Bands were quantified and results are reported as Mao A/β-actin ratios and shown as mean ± SE **(C)**.

**Figure 2:**
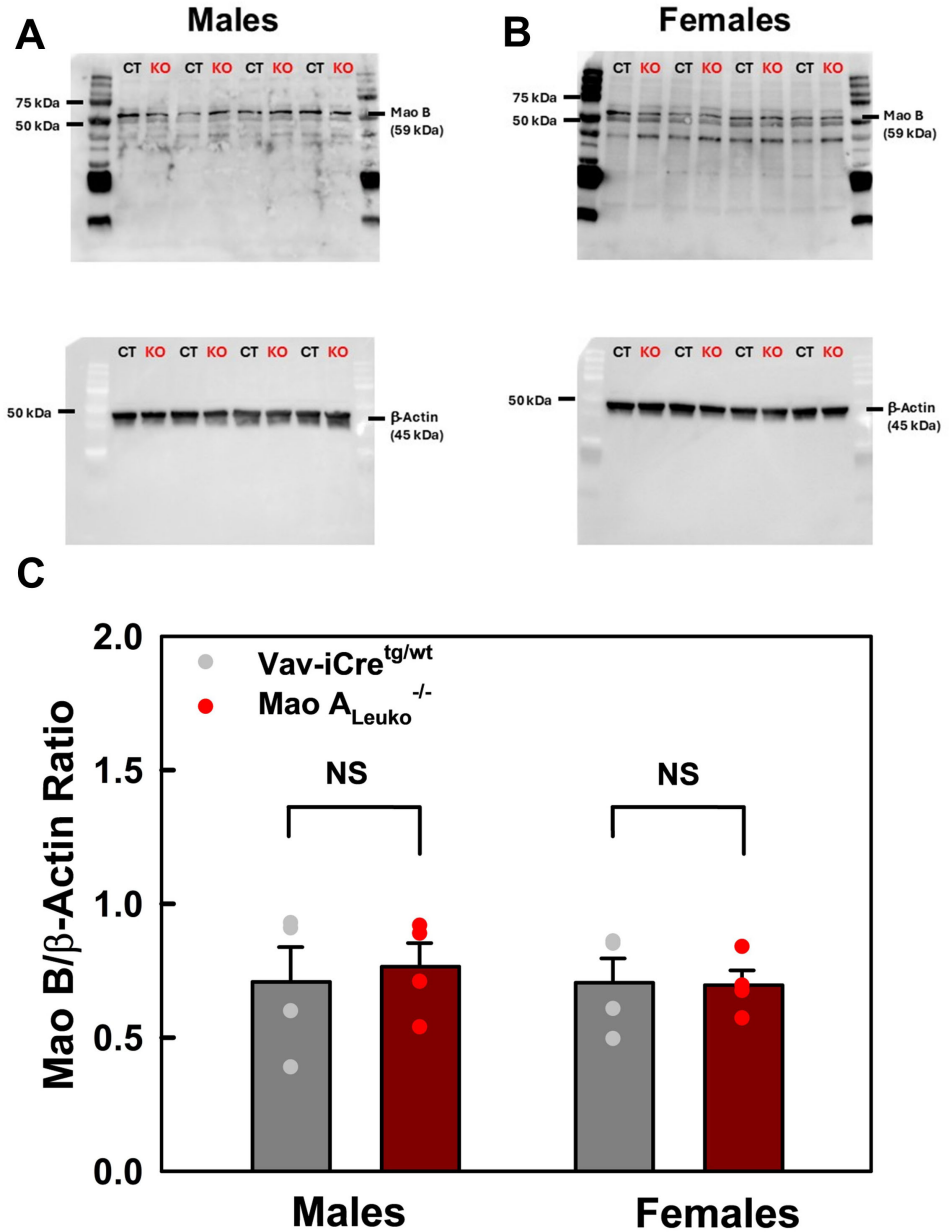
Mao B Expression Levels In White Blood Cells (WBC) Isolated From Vav-iCre^tg/wt^ And Mao A_Leuko_^-/-^ Mice. Blood samples were collected from the submandibular veins of male and female Vav-iCre^tg/wt^ (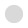, n = 4) and Mao A_Leuko_^-/-^ (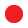, n = 4) mice, washed and treated with ice-cold red blood cell (RBC) lysis buffer to create a WBC pellet. Mao B expression in each of these WBC pellets was assessed by Western blot analysis **(A, B)**. The blot was then stripped and reprobed with β-actin as described in detail under “Materials and Methods”. Bands were quantified and results are reported as Mao A/β-actin ratios and shown as mean ± SE **(C)**.

To examine the role of hematopoietic Mao A in EAE, we, in collaboration with Applied StemCell, generated conditional Mao A knockout mice (Mao A^flox/flox^) mice using CRISPR/Cas9 in mice with a C57BL/6J genetic background and crossed them with Vav-iCre^tg/wt^ mice. In these mice, the deletion of Mao A is restricted to hematopoietic cells, which include all nucleated blood cells, i.e. leukocytes. We will therefore refer to these mice as Mao A_Leuko_^-/-^ mice. To assess the efficacy of Cre-mediated gene deletion in Mao A_Leuko_^-/-^ mice, we isolated white blood cells from four Mao A_Leuko_^-/-^ mice and four Vav-iCre^tg/wt^ control mice prior to EAE induction (day 0) and measured Mao A protein expression levels by Western blot analysis. Mao A protein levels were suppressed by 90% in male and by 91% in female Mao A_Leuko_^-/-^ mice compared to WBC from the respective Vav-iCre^tg/wt^ control mice (**Figure 1**). Considering that female mice carry two Mao A alleles and males only one, it was surprising to find that both male and female Vav-iCre^tg/wt^ control mice showed practically identical Mao A protein expression levels in WBC (**Figures 1A–C**). Deletion of Mao A in hematopoietic cells did not affect Mao B expression in WBC from either male or female mice (**Figures 2A–C**), indicating that Mao B did not compensate for loss of Mao A activity.

**Figure 3:**
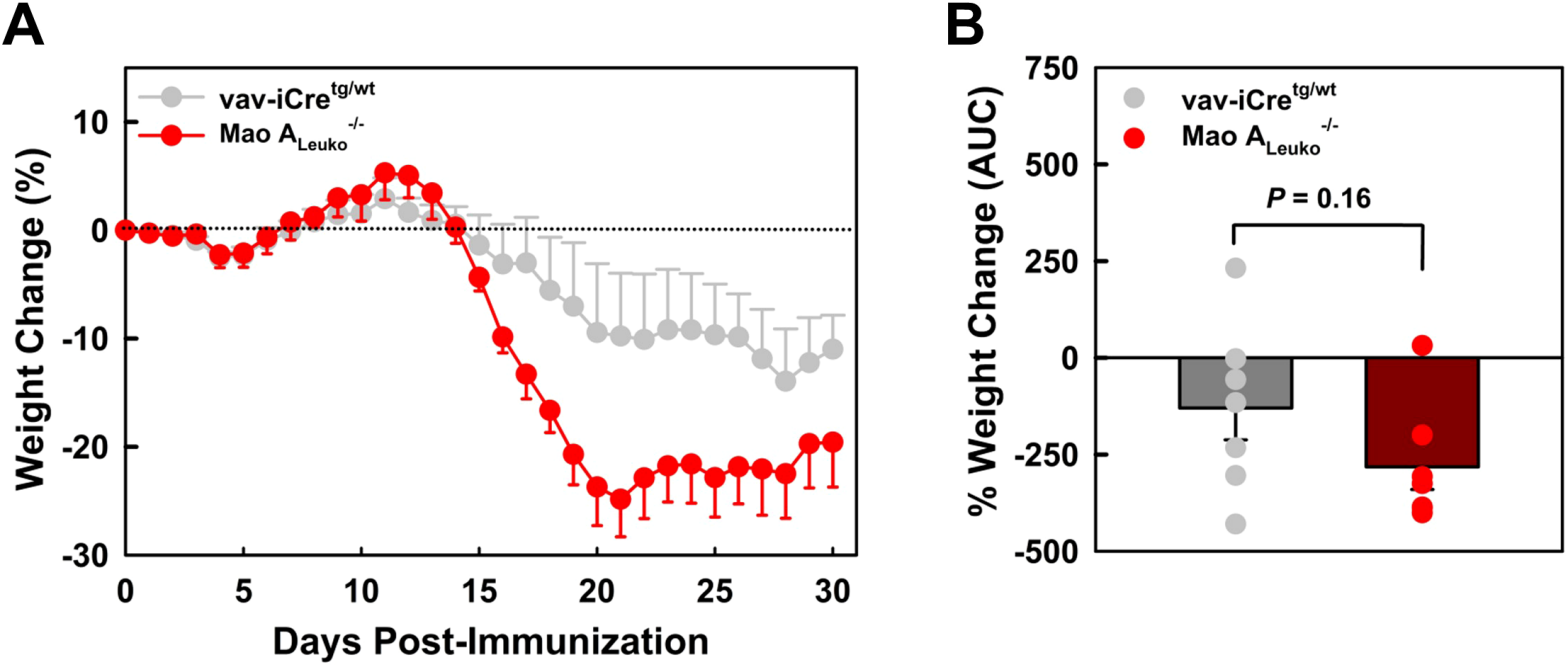
Hematopoietic Mao A Deficiency Increases Weight Loss Associated With EAE. Female Vav-iCre^tg/wt^ (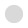, n = 7) and Mao A_Leuko_^-/-^ mice (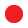, n = 7), were switched to a low-calorie MD and EAE was actively induced with a subcutaneous injection of mouse MOG_35–55_ emulsified in complete Freund’s adjuvant and two i.p. injections with 200 ng of PTX each at 2 h and 24 h after the MOG_35–55_/CFA injection. Weights were recorded daily for 30 days post-immunization **(A)** and changes in body weight were calculated as AUC **(B)**. Results are expressed as means ± SE.

Female mice maintained on an MD and immunized with MOG_35–55_ began losing weight on day 14 post-immunization and their weight loss peaked on day 28 with a mean weight loss of 14% (**Figure 3A**). Mao A_Leuko_^-/-^ also began losing weight on day 14 post-immunization, but their weight loss accelerated markedly compared to Vav-iCre^tg/wt^ mice and by day 21 reached its maximum at a mean loss of 22.5% of the original body weight (**Figure 3A**). The average cumulative weight loss was 2.2-fold greater in Mao A_Leuko_^-/-^ compared to Vav-iCre^tg/wt^ control (**Figure 3B**), but the difference between the means did not reach statistical significance. This finding suggested that contrary to our original hypothesis, loss of Mao A activity in leukocytes worsens EAE severity in female mice.

### 3.2 Hematopoietic Mao A deficiency increases EAE disease and ataxia severity

**Figure 4:**
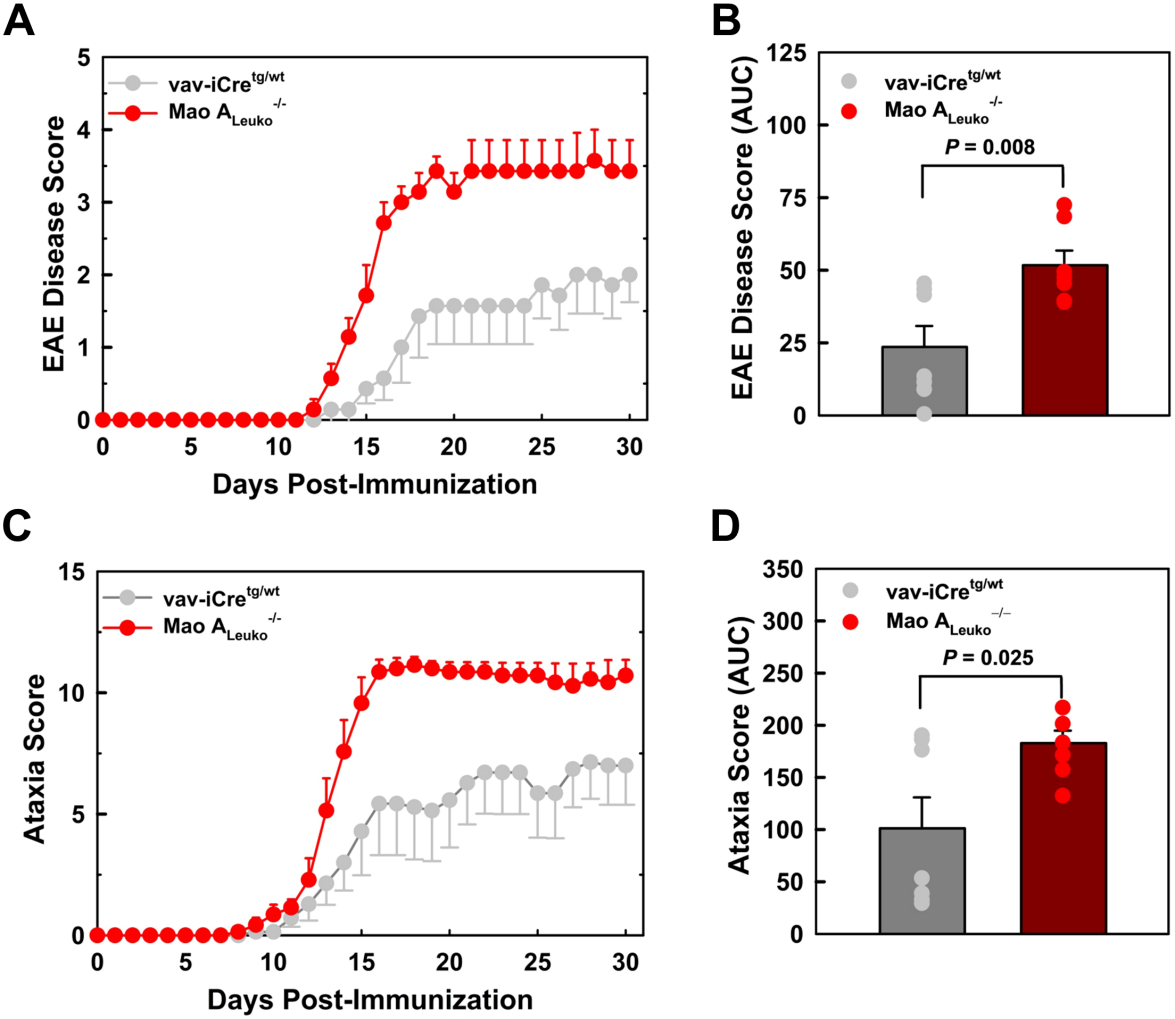
Hematopoietic Mao A Deficiency Increases EAE Disease and Ataxia Severity. EAE was actively induced in female Vav-iCre^tg/wt^ (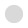, n = 7) and Mao A_Leuko_^-/-^ mice (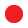, n = 7), as described in **Figure 3**. Clinical disease course was monitored daily and EAE disease **(A)** and ataxia severity **(C)** were scored for 30 days post-immunization, as described under “Materials and Methods”. Cumulative EAE disease and ataxia severity was calculated for each mouse as AUC **(B, D)**. Results are expressed as means ± SE. (n=7 per group).

MOG_35-55_-immunized EAE female mice in both groups began showing symptoms of EAE around day 13 post-immunization (**Figure 4A**). Disease severity increased rapidly thereafter and for the Vav-iCre^tg/wt^ control group peaked on day 19 at a mean score of 1.6, whereas the mean score for the Mao A_Leuko_^-/-^ group reached 3.4, after which both curves plateaued. By day 30, the differences between the mean scores for both groups were still 1.4. The cumulative increase in EAE severity, measured as the area under the curve (AUC), was 2.2-fold higher in the Mao A_Leuko_^-/-^ group than Vav-iCre^tg/wt^ control mice (**Figure 4B**), suggesting that Mao A in leukocytes suppresses EAE development and severity.

The rapid increase in the EAE disease score for Mao A_Leuko_^-/-^ was mirrored by the combined ataxia score, although the onset of visual physical impairment was already observed around day 10 post-immunization (**Figure 4C**). Mean ataxia scores in the Vav-iCre^tg/wt^ control only reached 5 on day 16 before the increase in the mean score slowed significantly. In contrast, on the same day the mean scores for the Mao A_Leuko_^-/-^ group peaked at 11. The mean scores for the Mao A_Leuko_^-/-^ group plateaued thereafter. The cumulative increase in ataxia severity was 1.8-fold higher in the Mao A_Leuko_^-/-^ group than Vav-iCre^tg/wt^ control mice (**Figure 4D**), confirming the protective role of leukocyte Mao A in EAE.

**Figure 5:**
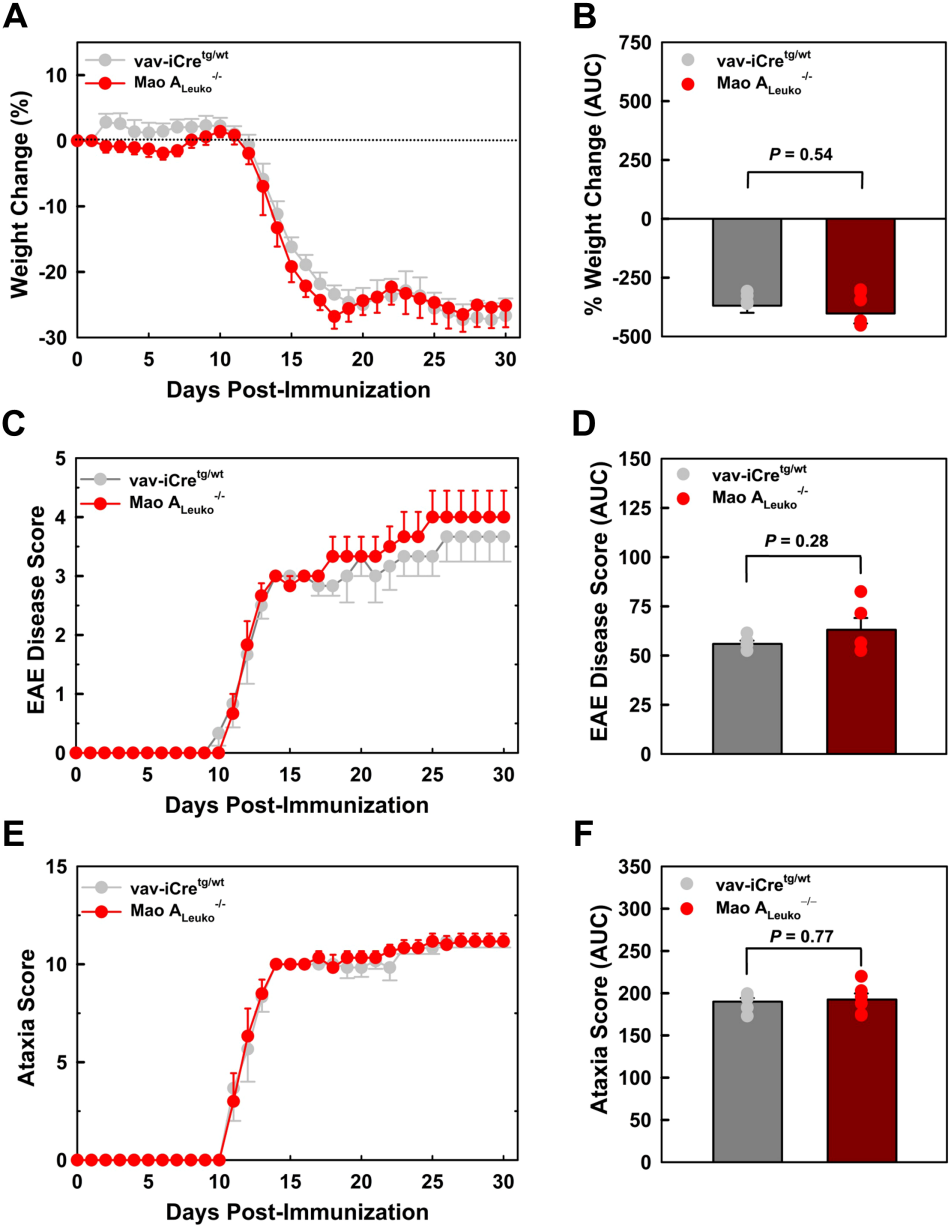
Hematopoietic Mao A Deficiency Impacted Neither Weight Loss nor the Severity of EAE Disease and Ataxia in Male EAE Mice. Male Vav-iCre^tg/wt^ (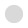, n = 6) and Mao A_Leuko_^-/-^ mice (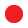, n = 6), were maintained on an MD before EAE was actively induced by subcutaneous injection with mouse MOG_35–55_ emulsified in complete Freund’s adjuvant and for 30 days after immunization. Daily, weights were recorded **(A, B)**, clinical disease course monitored, EAE **(C, D)** and ataxia severity **(E, F)** were scored for 30 days post-immunization, as described under “Materials and Methods”. Results are expressed as means ± SE.

In contrast, we observed no difference in EAE-induced weight changes between male Mao A_Leuko_^-/-^ mice and Vav-iCre^tg/wt^ controls with EAE (**Figures 5A, B**). Male mice developed EAE and ataxia, but we observed no significant differences in disease severity between Vav-iCre^tg/wt^ controls and Mao A_Leuko_^-/-^ (**Figures 5C–F**), demonstrating that in male mice, leukocyte Mao A is not involved in either the onset or the development of EAE. However, it is important to note that the EAE disease courses of Vav-iCre^tg/wt^ control mice differed significantly between males and females (**Figures 4A, B** versus **Figures 5C, D**), males showing a 2.4-fold greater cumulative severity (**Figures 4B, D**; grey bars, *P* < 0.004). In contrast, in the widely used MOG_35-55_-induced EAE model, male and female C57BL/6 mice show nearly identical clinical disease courses and cumulative severity (50–52).

### 3.3 Hematopoietic Mao A deficiency enhances inflammation and accelerates demyelination within EAE lesions

**Figure 6:**
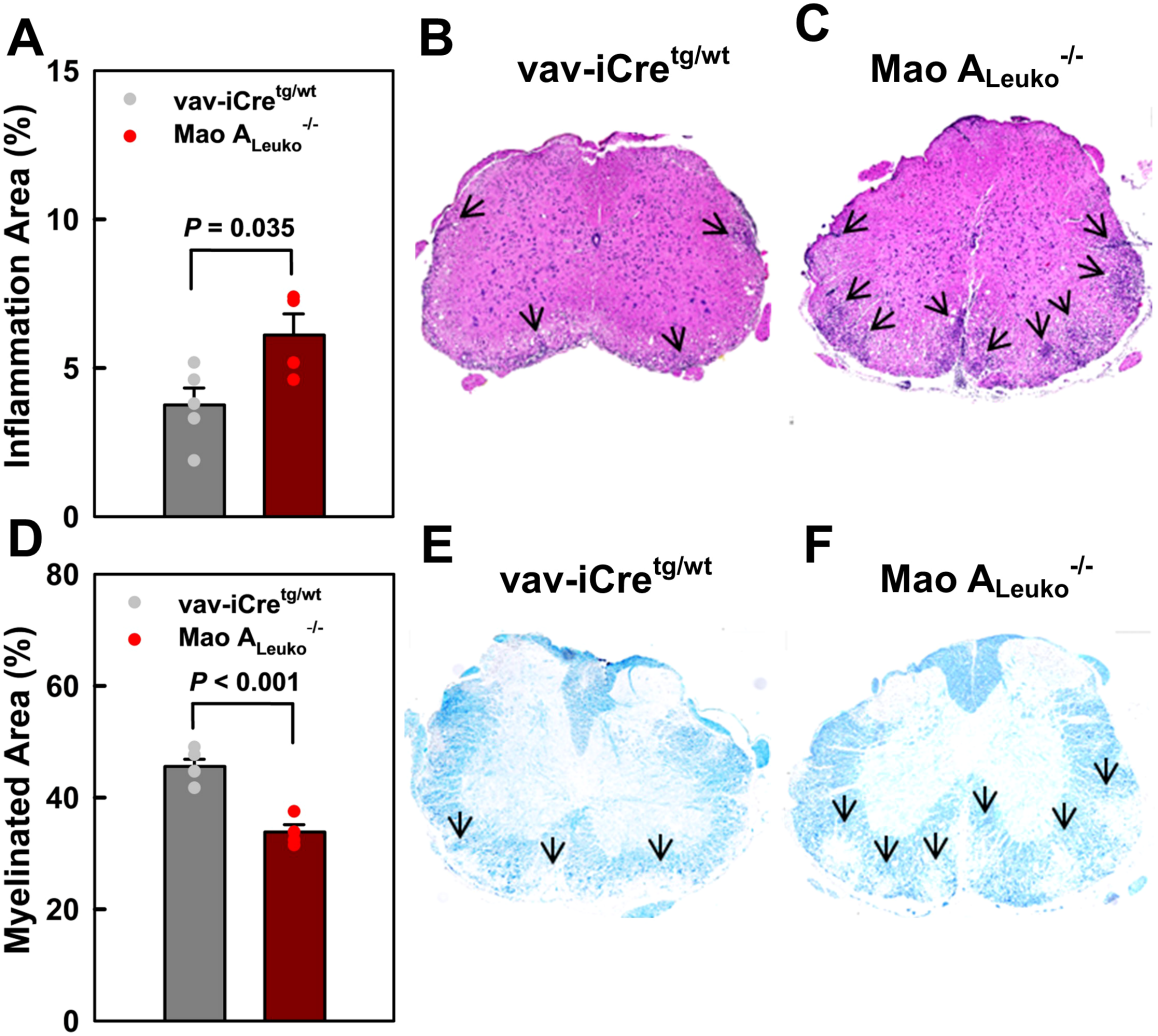
Hematopoietic Mao A Deficiency Enhances Inflammation And Accelerates Demyelination in the Lumbar Spinal Cords Of EAE Mice. Three lumbar spinal cord sections from randomly selected female Vav-iCre^tg/wt^ (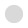, n = 5) and Mao A_Leuko_^-/-^ mice (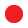, n = 4), were stained with H&E **(A–C)** or Luxol Fast Blue **(D–F)**. H&E sections were scored in a blinded fashion for their level of inflammation as detailed under “Materials and Methods”. Mean inflammation scores for each mouse are shown in panel A. Myelination was assessed as % area of the section that stained positive for Luxol Fast Blue (LFB). The mean % LFB-positive area of three sections was determined for each mouse and is shown in panel D. Results are expressed as means ± SE.

Histopathological examinations of sections collected from the lumbar spinal cord of all EAE mice revealed extensive leukocyte infiltration and inflammation in the tissues from Vav-iCre^tg/wt^ control mice, an indicator of ongoing inflammation (**Figures 6A, B**). Mao A deficiency in leukocytes of EAE mice increased leukocyte infiltration by 63%, indicating increased inflammation within the lumbar spinal cords (**Figures 6A, C**). Importantly, lumbar spinal cord sections from Mao A_Leuko_^-/-^ EAE mice also showed a 26% reduction in myelinated areas compared with sections obtained from the same area in Vav-iCre^tg/wt^ control mice with EAE, suggesting that loss of Mao A activity in leukocytes accelerates demyelination in EAE mice (**Figures 6D–F**).

### 3.4 Hematopoietic Mao A deficiency exacerbates the activation of macrophages and microglia in the CNS of EAE mice

**Figure 7:**
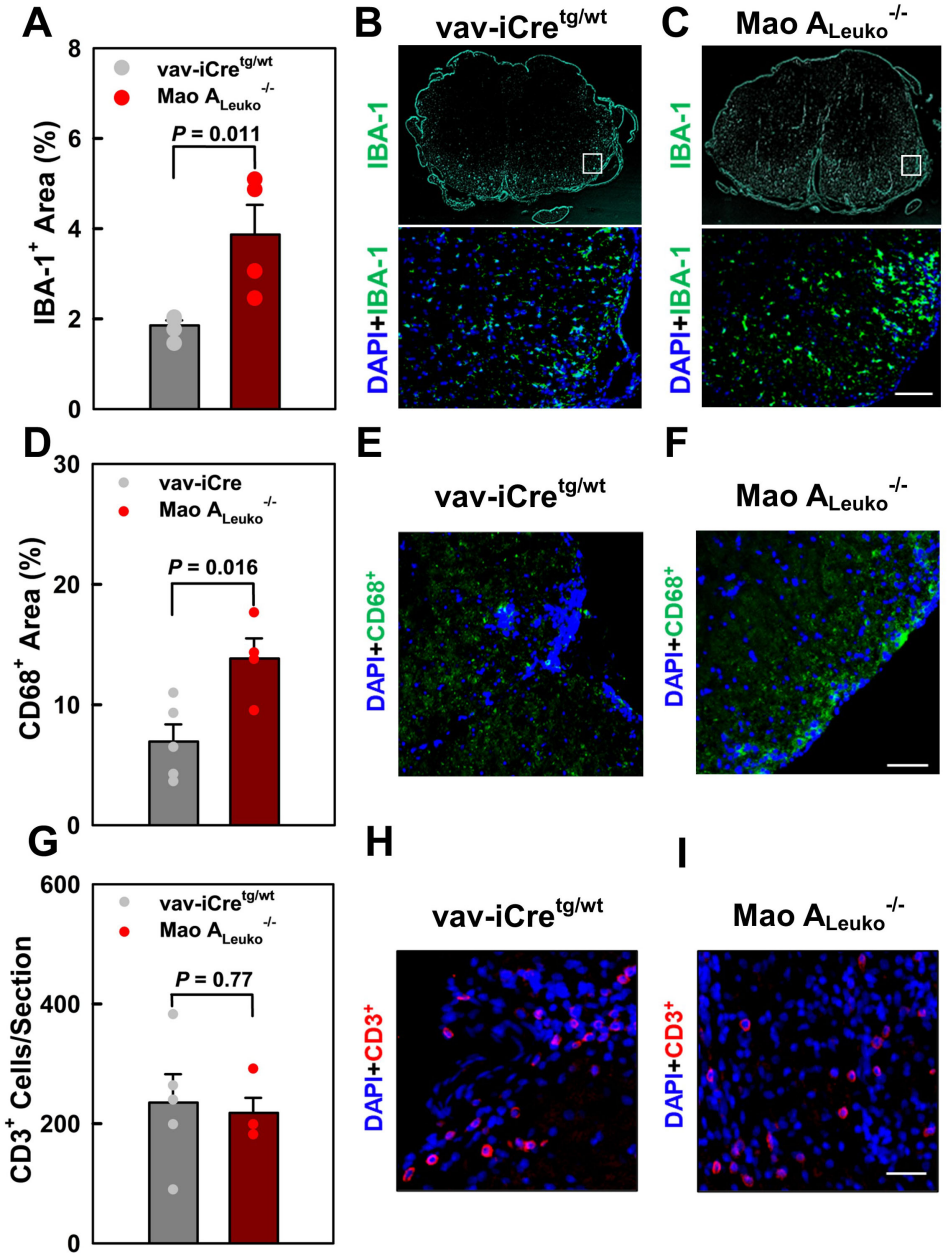
Hematopoietic Mao A Deficiency Exacerbates Microglia/Macrophage Recruitment and Activation, but Does not Alter T Cell Content in the Lumbar Spinal Cords of EAE Mice. Three lumbar spinal cord sections from randomly selected female Vav-iCre^tg/wt^ (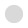, n = 5) and Mao A_Leuko_^-/-^ mice (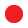, n = 4), were subjected to immunofluorescence staining and image analysis. Sections were blocked and treated with primary antibodies directed against CD68 **(A–C)**, Iba-1 **(D–F)** and CD3 **(G–I)** followed by Alexa Fluor Plus 488 (Green) or Alexa Fluor 594 (Red)-conjugated secondary antibodies, and finally counter-stained with DAPI (Blue), as described under “Materials and Methods”. Macrophage/Microglia recruitment and accumulation was assessed as % area of the section that stained positive for CD68 **(A)**, activation of microglia and macrophages was assessed as % area of the section that stained positive for IBA-1 **(D)**, and T cell content was determined as the number of CD3^+^ cells per section **(G)**. Results are expressed as means ± SE. Scale bar = 100 μm.

Previous studies have shown that monocyte-derived macrophages (MDM) contribute to the demyelination and inflammation in EAE (53–55). To determine the impact of hematopoietic Mao A deletion on the activation state of MDM and microglia and other resident macrophages in the CNS during EAE, we assessed in spinal cord sections from Vav-Cre^tg/wt^ and Mao A_Leuko_^-/-^ mice the expression of IBA-1, a marker specific for macrophages and microglia, which is upregulated during cell activation (56–58). In sections from Mao A_Leuko_^-/-^ mice, IBA-1 immunostaining revealed an increase in IBA-1^+^ cells (**Figures 7A–C**), suggesting microglia/macrophage activation and, potentially, increased infiltration of the CNS by MDM. The latter was confirmed by immunostaining with antibodies directed against CD68 (**Figures 7D–F**), a marker for lysosomal activity in macrophages and microglia.

EAE is considered a T cell–mediated disease (59) and our immunohistochemical analysis of the lumbar spinal cord a robust infiltration of CD3^+^ T-cells (**Figures 7H, I**). However, we did not observe any significant difference in T-cells levels between Mao A_Leuko_^-/-^ and Vav-i Cre^tg/wt^ EAE mice (**Figure 7G**). This finding suggests that the exacerbated disease phenotype in female Mao A_Leuko_^-/-^ is not driven by increased recruitment of T cells into the CNS.

## 4 Discussion

The goal of this study was to explore the role of Mao A in immune cells on the onset, progression and severity of EAE, a murine model of human multiple sclerosis (16, 17). We found that deletion of Mao A in hematopoietic cells, which includes circulating immune cells, increased the severity of EAE diseases and exacerbated motor deficits, but only in female mice. Hematopoietic Mao A deficiency in male mice did not affect EAE disease severity, motor deficits or weight loss. However, the severity of both EAE and ataxia in males mice, which or without Mao A deficiency (**Figure 5**), was very similar to the disease severity and degree of motor deficits we observed in female EAE mice with hematopoietic Mao A deletion (**Figure 4**). These findings suggest that Mao A in immune cells protects female mice against EAE. Why immune cell Mao A does not convey a similar protection to male mice with EAE is unclear.

One possible mechanism that may explain this sex difference is that Mao A is involved in estrogen signaling and thus in mediating the protective properties of estrogen against neuroinflammation and oxidative stress (60–62). This hypothesis appears to contradict reports that claim that in women estrogen suppresses Mao A expression and activity (63–65). However, this does not appear to be the case for female mice. We recently reported in a mouse model of diet-induced obesity (DIO), that Mao A protein expression in adipose tissue isolated from female mice is actually higher than in males, whereas Mao A activity in adipose tissue was not statistically different between males and females (66). These findings suggest that at least in mice, estrogen does not suppress Mao A activity. In the same study, we also reported that Mao A deficiency in polarized BMDM amplifies their expression of activation state-specific marker genes, suggesting that at least in BMDM, Mao A regulates macrophage signaling and activation (66). The increased expression of the macrophage/microglia-specific cell activation marker IBA-1 (56, 57, 65) we observed in lesions of female EAE mice with hematopoietic Mao A deficiency (**Figures 7A–C**) appears to support this hypothesis. We went on to speculate that Mao A-derived H_2_O_2_ mediates the effects of Mao A on cell signaling by targeting redox sensitive signaling molecules and transcription factors. Whether this regulatory role of Mao A in cell signaling in the EAE model demonstrated that recruitment of MDM into the CNS, is essential for demyelination to occur and for the associated clinical manifestations (53, 67–69). It is therefore likely that macrophages are primarily responsible for the protective role(s) of Mao A in female EAE mice.

Microglia and MDM express estrogen receptors (ERα/ERβ/GPER1) and exhibit sex- and region-specific functional differences that shape neuroinflammatory responses (70, 71) (72). Estradiol signaling can directly modulate microglial maturation, phenotype, and cytokine programs. Given this synergistic relationship between microglia and gonadal hormones, it is not surprising that there are sex differences in microglial morphology and function. Therefore, the protective effect of Mao A in females may depend on estrogen-responsive signaling nodes that alter redox tone and inflammatory set-points.

Another complementary axis is noradrenergic control of microglia. Noradrenaline (NA), acting via β2-adrenergic receptors, polarizes microglia toward anti-inflammatory activation states (73). Pharmacologic activation of β2-AR in microglia reduces pro-inflammatory signaling and attenuates neuropathic hypersensitivity *in vivo* (74). In the context of neurodegenerative pathologies, dampening NA signaling or β-adrenergic tone has been shown to aggravate microgliosis and neuroinflammation (73). Given Mao A’s role in NA catabolism, hematopoietic Mao A deletion could alter peripheral monoamine handling and the immune-CNS crosstalk. Coupled with sex-dependent adrenergic receptor expression and estrogenic modulation, these layers of signaling may collectively explain the female-restricted sensitivity to loss of Mao A activity we observed in EAE mice.

One limitation of our study lies in the fact that we observed a difference in the EAE disease course and severity between male and female Vav-iCre^tg/wt^ control mice, whereas disease courses and cumulative severity reported for MOG_35-55_-induced EAE in male and female C57BL/6 mice, are very similar (50–52). At the present time, we do not know what the reason for this sex difference is. However, we suspect that the Vav-iCre cassette may be responsible for this discrepancy. The presence of the Vav-iCre transgene in female EAE mice appears to unmask a Mao A sensitivity, as loss of hematopoietic Mao A sensitizes female (but not male) Vav-iCre mice to EAE and consistently exacerbated disease severity, weight loss, and the neuropathology, whereas no such effects were observed in male EAE mice.

Furthermore, the Vav-iCre transgene in the mouse strain 018968 from The Jackson Laboratory has been shown to be active in the testes of male mice and can result in Cre-mediated recombination in germ cells. Therefore, if Cre^+^ males are used in crosses, it is possible that unintended germline deletion of the floxed allele may occur in all tissues of the offspring, rather than a tissue-specific deletion. Alternatively, Vav-Cre/Vav-iCre strains have also been reported to show Cre expression in endothelial cells (75, 76). While this is a general issue with hematopoietic drivers, this could theoretically lead to skewed data in male versus female bone marrow environments.

In summary, we identified a previously unrecognized role for Mao A in the development and progression of EAE and possibly MS. Further, we identified a novel mechanism that may contribute to sex differences in pathologies involving macrophages, including chronic (neuro)inflammatory diseases. Therapeutic strategies that result in the induction of Mao A in macrophages may therefore represent a novel (adjunct) therapy for women with or at risk for MS.

## Data Availability

The raw data supporting the conclusions of this article will be made available by the authors, without undue reservation.
